# Using artificial intelligence to develop a measure of orthopaedic treatment success from clinical notes

**DOI:** 10.3389/fdgth.2025.1523953

**Published:** 2025-04-24

**Authors:** Sarah B. Floyd, Ahmed G. Almeldien, D. Hudson Smith, Benjamin Judkins, Claire E. Krohn, Zachary Cole Reynolds, Kyle Jeray, Jihad S. Obeid

**Affiliations:** ^1^ Department of Public Health Sciences, Clemson University, Clemson, SC, United States; ^2^ Biomedical Data Science and Informatics Program, Clemson University, Clemson, SC, United States; ^3^ Department of Mathematical and Statistical Sciences, Clemson University, Clemson, SC, United States; ^4^ Department of Orthopaedic Surgery, Prisma Health, Greenville, SC, United States; ^5^ Department of Public Health Sciences, Medical University of South Carolina, Charleston, SC, United States

**Keywords:** proximal humerus fractures, artificial intelligence, treatment outcomes, clinical text, natural language processing, electronic health record system

## Abstract

**Introduction:**

A readily available outcome measure that reflects the success of a patient's treatment is needed to demonstrate the value of orthopaedic interventions. Patient-reported outcome measures (PROMs) are survey-based instruments that collect joint-specific and general health perceptions on symptoms, functioning, and health-related quality of life. PROMs are considered the gold standard outcome measure in orthopaedic medicine, but their use is limited in real-world practice due to challenges with technology integration, the pace of clinic workflows, and patient compliance. Clinical notes generated during each encounter patients have with their physician contain rich information on current disease symptoms, rehabilitation progress, and unexpected complications. Artificial intelligence (AI) methods can be used to identify phrases of treatment success or failure captured in clinical notes and discern an indicator of treatment success for orthopaedic patients.

**Methods:**

This was a cross-sectional analysis of clinical notes from a sample of patients with an acute shoulder injury. The study included adult patients presenting to a Level-1 Trauma Center and regional health system for an acute Proximal Humerus Fracture (PHF) between January 1, 2019 and December 31, 2021. We used the progress note from the office visit for PHF-related care (ICD10: S42.2XXX) or shoulder pain (ICD10: M45.2XXX) closest to 1-year after the injury date. Clinical notes were reviewed by an orthopaedic resident and labeled as treatment success or failure. A structured comparative analysis of classifiers including both machine and deep learning algorithms was performed.

**Results:**

The final sample included 868 clinical notes from patients treated by 123 physicians across 35 departments within one regional health system. The study sample was stratified into 465 notes labeled as treatment success and 403 labeled as treatment failure. The Bio-ClinicalBERT model had the highest performance of 87% accuracy (AUC = 0.87 ± 0.04) in correctly distinguishing between treatment success and failure notes.

**Discussion:**

Our results suggest that text classifiers applied to clinical notes are capable of differentiating patients with successful treatment outcomes with high levels of accuracy. This finding is encouraging, signaling that routinely collected clinical note content may serve as a data source to develop an outcome measure for orthopaedic patients.

## Introduction

A readily available outcome measure that reflects the success of a patients' treatment is needed to demonstrate the value of orthopaedic interventions (1–4). PROMs are viewed as the gold standard outcome measure in orthopaedics (5, 6), and could be used to track improvements in patient status over time to assess whether a patient achieved their treatment goals (1, 7, 8). However, PROM collection in real-world practice has proven difficult due to challenges with technology integration, data collection disruptions to clinic workflows, and low patient compliance completing PROM questionnaires (9, 10). Most orthopaedic practices in the United States view the collection of PROMs as time consuming, cumbersome, and costly (4, 11), and it is estimated that only half of orthopaedic practices collect PROMs (9, 12). Without widespread availability of PROMs, evaluating the success of orthopaedic treatments has been limited to process-based measures and end points such as survival and surgical complications, which can readily be found in structured fields in administrative data (4). Unfortunately, these data points paint an incomplete picture of a patient's medical experience and whether treatment was successful in achieving a patient's prioritized outcome goals (e.g., reduced pain, improved range of motion, return to sports or work, etc.) (13–16). A crucial step to demonstrating if orthopaedic care achieves meaningful patient-specific improvement is to have outcome data available that measure if treatment was successful in achieving the outcome goals prioritized by patients.

Unstructured clinical notes represent untapped potential for evaluating the success of orthopaedic treatments for individual patients (17–24). Clinical notes are generated for each encounter patients have with their physician. These notes contain rich information on current disease symptoms, rehabilitation progress, and unexpected complications, and captures an essence of the patient state that is not stored elsewhere in the Electronic Health Record (EHR) (19, 25–30). However, because these notes are in an unstructured format, they have not historically been thought of as a useful data source to develop consistent measures of patient treatment success. Applications of artificial intelligence (AI) and natural language processing (NLP) methods in healthcare are rapidly expanding, and have been used to identify similar patient groups with a defined set of clinical markers and symptoms, automate patient cohort selection, and develop predictive models (17, 25, 31–33). In orthopaedic medicine, NLP applications are been being deployed to solve a range of orthopaedic-related problems (34–36). We proposed that NLP methods can be employed to identify phrases denoting treatment success or failure recorded in clinical notes, thereby discerning indicators of treatment success for orthopaedic patients.

Clinical notes are typically documented using a subjective, objective, assessment and plan (SOAP) format, where the subjective section of the progress note reflects the patient's story (the interim history since the last visit), told to and interpreted by the physician (28). The rest of the unstructured SOAP notes include physical findings (objective findings), medical reasoning (assessment), and patient care (the plan) and (37–39) reveal distinct trajectories of patient outcomes after treatment (38, 40). In successful cases the progress note documents the degree of improvement or relief experienced and reported by patients to their clinicians (30). Conversely when symptoms have not resolved, or when subsequent complications arise, these ongoing patient complaints and persistent treatment utilization are documented in the notes. Note content highlights symptoms and outcome dimensions, which are valued by patients and contribute to a patient's individual definition of success (41).

A patient-specific outcome measure is needed in orthopaedic medicine to assess if a patient's treatment goals were met and help demonstrate the value of orthopaedic treatments. The objective of this study was to develop a clinical text classifier, using different AI approaches, including traditional machine learning and deep learning, capable of identifying whether a patient achieved treatment success for a sample of patients receiving follow-up care for an acute proximal humerus fracture. Proximal humerus fracture (PHF) was chosen as a use case for testing this method of outcome generation because many different treatment options can be used for this injury and better outcome data are needed to guide future clinical decision-making for this condition (42–45). To that end, we began by exploring the feasibility of generating an outcome measure from clinical notes by evaluating various AI-based text classifiers for patients treated for a PHF.

## Materials and methods

### Study sample

This was a cross-sectional analysis of clinical notes from a sample of patients with an acute PHF. The study included adult patients presenting to a Level-1 Trauma Center regional health system for an acute PHF between January 1, 2019 and December 31, 2021. The first visit for a patient for PHF during the study period was defined as the index visit. We then identified all health system encounters (hospital encounters, office visits, etc.) with a diagnosis of PHF or shoulder pain from the index PHF visit to 365 days after the index PHF visit and extracted the clinical note from the office visit for PHF-related care (ICD10: S42.2XXX) or shoulder pain (ICD10: M45.2XXX) closest to 1-year after the injury date. This resulted in one representative note per person. Patients were excluded from the study if they were less than 18 years of age, did not have at least one office visit with a diagnosis of PHF or shoulder pain that occurred 45 days or more days after the index visit, or if their last office visit was less than 500 characters, since smaller notes did not have the necessary sections for outcome information. We found that less than 1% of the eligible sample had a note length less than 500 characters. A minimum of 45 days after index was used as this is the minimal time needed for healing of a PHF, prior to which, treatment success cannot be assessed. We randomly sampled 1,000 patients meeting these inclusion criteria, and after physician review of the sample, a total of 868 notes were used for this study. Additional exclusions after physician review included notes from patients receiving care for bilateral PHFs or notes that did not reflect care for PHF. This study was approved by the Prisma Health Institutional Review Board.

### Data labeling process

The University of South Carolina Patient Engagement Studio brings together patients and caregivers, community groups, health system innovators, clinicians, and academic researchers to produce meaningful research that advances health research outcomes. The PES membership includes over 100 patients with diverse backgrounds and clinical experiences from across the United States trained to provide feedback and collaborate with research teams (46, 47). The first author (S.F.) led three Patient Engagement Studio (PES) sessions to involve patients in the development of the definitions of treatment success. There is no one standard acceptable definition of treatment success, as it varies based upon patient lifestyle and desired goals (4, 48). However, generally, the goal of orthopaedic treatment is to restore the function of the joint, minimize pain and maximize quality of life for patients after the injury. Together, the PES members and research team defined four outcome states and associated labels that described the range of positive and negative outcomes that could follow care for PHF. The outcome states, definitions, and note text indicators can be found in Table 1.

**Table 1:** Outcome labels and corresponding definition and indicators.

Outcome state	Definition	Indicators of outcome state found in clinical notes
Treatment success	Treatment success occurs when a patient is able to resume desired activities, has a sufficient range of motion, and is in minimal/mild or no pain. After PHF it is possible for there to be some lingering motion limitations (patient may never return to 100%) or minimal pain, but these issues should not require ongoing treatment or be prohibitive to their desired lifestyle or daily activities.	• Radiographic healing • Making good progress/improvements with current treatment or stopping treatment • Patient returns to work or play. • No major complaints documented. • Only follow-up as needed
Improvement of condition	Improvement occurs when there is a record of some levels of pain or functional problems that are somewhat prohibitive to the patient's desired activities, but improvement is occurring. In these situations, physicians may continue to monitor patients, but do not alter care or treatment courses.	• Radiographic healing or signs of healing occurring. • Moderate loss of function or pain which interferes with desired activities, but no change in treatment. • Ongoing treatment and monitoring progress • Return in 2–6 weeks for repeat x-rays and re-check
Deterioration of condition	Deterioration occurs when there is a record of some levels of pain or functional problems that are becoming more prohibitive to the patient's desired activities. No real improvements are occurring, and physicians may escalate or alter care or treatment courses.	• Negative radiographic changes observed. • Moderate loss of function or pain which interferes with desired activities requiring a change in treatment. • Initiating or continuing treatment and monitoring progress. • Return in 2–6 weeks for repeat x-rays and re-check.
Treatment Failure	Treatment failure occurs when the patient is experiencing significant pain or limitations and requires subsequent fracture-related care. Failing is occurring when patients are unable to resume desired activities and may include fracture sequelae, complications, or nonunion.	• Ongoing, persistent treatment (injections, surgeries) for symptoms related to PHF. • Unrelenting pain • Surgical complications • Loss of significant motion • Extreme pain • Fracture related sequelae (e.g., avascular necrosis)

Four orthopaedic residents, each with a minimum of 2 years of experience in orthopaedic medicine participated in the labeling of the clinical notes. Each orthopaedic resident received a 1-h training session on the note labels and definitions. Residents were instructed to label each note based on the outcome state reflected in the current visit and documented in the note. In addition to the outcome labels, orthopaedic residents had the option to select “Indeterminant—not enough content to label” or flag a note for expert review. We assigned overlap in note review subsets, so that agreement could be assessed across residents. An attending orthopaedic surgeon, the Chair of Department of Orthopaedic Surgery, served as the final arbitrator when discordance occurred between residents’ labels or when expert review was requested. Each label provided by the Chair was viewed as the final label for that note.

REDCap (49) was used to organize and store notes and labels. Notes were truncated to 10,000 words and HTML tags were added for formatting to aid in the review process. The final labeled dataset produced by the orthopaedic residents represented the gold standard dataset used for model development and validation. The four outcome labels were aggregated into a binary outcome representing treatment success or failure. Treatment success was represented by notes labeled as success. Failure was comprised of all remaining labels (improvement, deterioration and failure) as each of those notes had documentation of lingering, symptomatic problems requiring ongoing care. This was done to avoid class imbalance between the treatment success and failure classes, since our data set had many more cases in the treatment success category compared to the remaining three unfavorable outcome categories.

### Stratified data segmentation protocol

**Figure 1:**
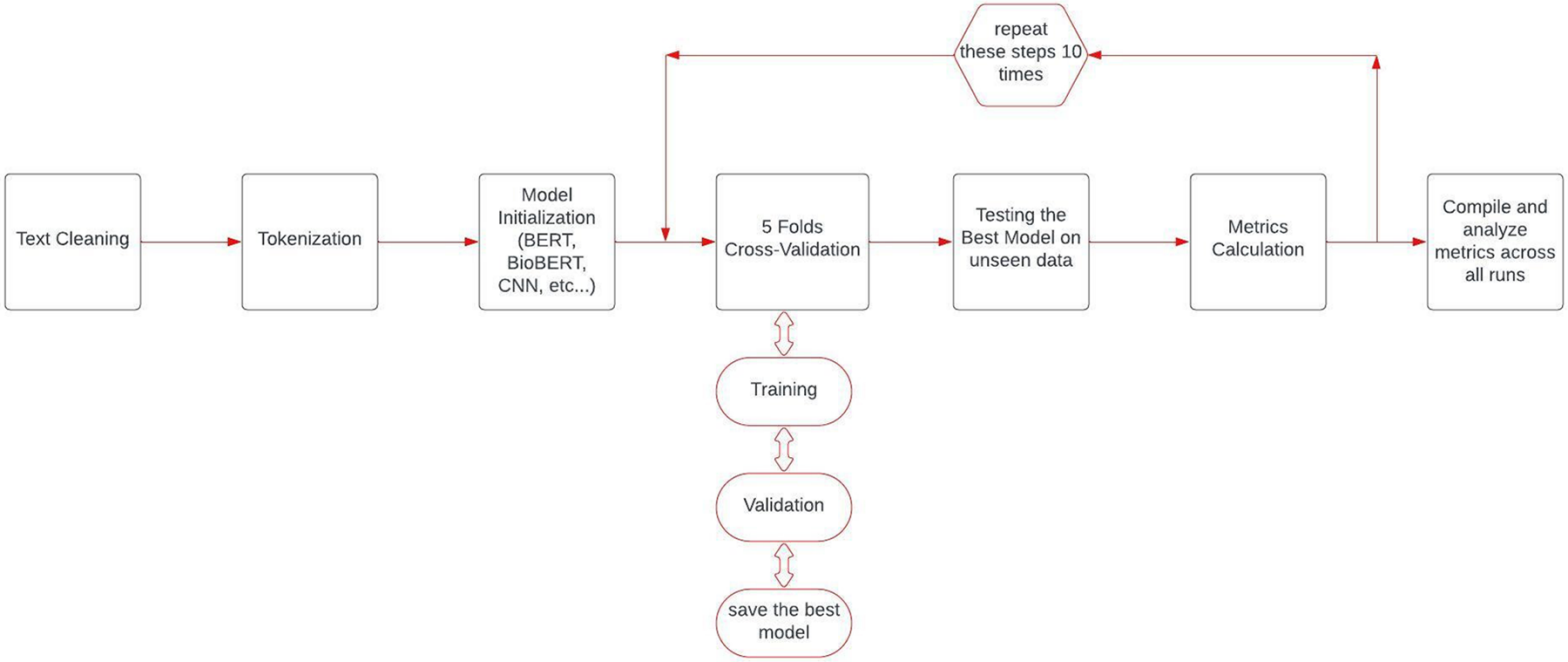
AI model training and evaluation pipeline for treatment success measure assessment.

We maintained a consistent data division strategy to ensure a fair and unbiased comparison across all models, so 90% of the data was allocated for model training, while the remaining unseen 10% was reserved for testing. Within these partitions, we preserved the proportion of outcome labels observed in the full labeled data set. We used a five-fold cross-validation strategy to robustly measure the generalizability of all models. Moreover, due to the relatively small number of cases, we repeated the testing cycle 10 times through train and test data partitioning (Figure 1). A critical aspect of our experimental design was ensuring fair model comparison by using identical data splits across all models within each run. For each of the ten experimental iterations, we created a single stratified train-test split that was subsequently used for all models being evaluated. This approach eliminates potential confounding effects from data partitioning and ensures that any observed performance differences can be attributed to the models themselves rather than to variations in training or testing data.

### Bag-of-words based models

We tested the performance of several baseline classifiers that utilize a bag-of-words approach, primarily to provide a reference point for comparing more advanced modeling techniques. For these traditional machine learning models, we employed GridSearchCV with 5-fold cross-validation to systematically search for optimal hyperparameters. The text data were vectorized using term frequency-inverse document frequency (TF-IDF) (50) with parameters set to ignore terms appearing in more than 90% of documents (max_df = 0.9), include terms appearing in at least 5 documents (min_df = 5), and incorporate both unigrams and bigrams [ngram_range = (1,2)]. Text preprocessing was consistent across all models, involving lowercase conversion, special character removal (except for periods, numbers, and alphabets), whitespace normalization, and lemmatization for words longer than three characters. This approach preserved medical abbreviations that might be clinically significant while reducing vocabulary size and standardizing word forms.

After applying TF-IDF vectorization, we tested five distinct machine learning classifiers: Logistic Regression, with an inverse regularization strength (C) equal to 0.1; Support Vector Machine with Linear Kernel (51); Random Forest model with 200 trees, with each tree having a maximum depth of 10 and using “entropy” as the criterion for measuring the quality of splits (52); Gradient Boosting Classifier with 200 boosting stages and a learning rate of 0.01, implying effectiveness in gradual learning over numerous iterations (53); and XGBClassifier with 100 estimators, a learning rate of 0.01, a maximum tree depth of 4, and a subsample ratio of 0.9, indicating a preference for moderate complexity and high sample coverage in training (54).

### Transformer-based classifiers

In this study, we adopted a family of transformer-based language models to analyze clinical note content, primarily because transformers utilize a multi-head self-attention mechanism that pinpoints how different words in a sequence relate to each other, even when they are far apart. This mechanism effectively captures non-linear contextual dependencies by allowing multiple “heads” to attend to different segments of the input in parallel, thereby highlighting important clinical cues that might not be locally adjacent. Of the transformer-based models (55), we used BERT (56), BioBERT (57), and Bio-ClinicalBERT (58), and to analyze note content. We selected BERT as our base architecture model due to its bidirectional training paradigm, which uses both preceding and succeeding words for context. Building upon BERT, BioBERT further refines the model with additional pre-training on domain-specific biomedical corpora, effectively incorporating specialized terminology. Similarly, Bio-ClinicalBERT further refines BioBERT by fine-tuning on the Medical Information Mart for Intensive Care (MIMIC-III) (59) clinical notes dataset to augment its capability in deciphering medical terminology and understanding complex clinical semantics. Each of our transformer-based models accepted a maximum of 512 tokens. We selected the last 512 tokens instead of the first 512 tokens based on our observation that later parts of the note typically contained the most-clinically relevant information about ongoing symptoms and necessary treatment or discharge from care.

For these transformer-based models, we employed a consistent training approach. Each model was trained for 8 epochs with a batch size of 8, using the AdamW optimizer (71) with a learning rate of 3 × 10^−5^ and epsilon of 1 × 10^−8^. To optimize the learning rate schedule, we implemented a cosine annealing learning rate scheduler that gradually reduced the learning rate from the initial value to zero throughout the training process. We applied gradient clipping with a maximum norm of 1.0 to prevent exploding gradients during backpropagation. This technique helped stabilize the training process, particularly important for the fine-tuning of pre-trained language models on domain-specific data. The transformer-based models processed our common data splits through their respective tokenization pipelines, ensuring consistent evaluation across experimental runs.

### Convolutional neural network classifier

We also tested the performance of Convolutional Neural Network (CNN)-based classifiers for textual data, primarily because CNNs excel at extracting localized patterns from sequences—such as n-gram features—by sliding convolutional filters over the embedded text (60). We first converted the text into integer sequences and right padded these to a fixed sequence length (61, 62). Our CNN architecture was specifically designed for text classification, featuring an embedding layer followed by multiple parallel convolutional layers with varying filter sizes (3–12) to capture different n-gram patterns in the text. Each convolutional layer utilized 200 filters and was connected to an adaptive max pooling layer. The architecture incorporated a three-layer fully connected network (200 units → 100 units → 1 unit) with ReLU activations and dropout (rate = 0.5) for regularization. The CNN model was trained for up to 50 epochs with early stopping (patience = 5) using the Adam optimizer with an initial learning rate of 0.001 and a step learning rate scheduler (step size = 5, gamma = 0.1). Unlike the transformer models, the CNN implementation used a custom tokenizer and a batch size of 16, which resulted in a different processing pipeline for the same underlying data splits.

### Statistical analysis and model evaluation

Fleiss' kappa statistics were used to assess the degree of agreement in note labels between orthopaedic residents. We used the benchmarks for agreement measures for categorical data as described by Landis and Koch, where 0.00–0.20, 0.21–0.40, 0.41–0.60, 0.61–0.80, and 0.81–1.00 indicate poor, fair, moderate, substantial, and almost perfect agreement, respectively (63). Patient characteristics including patient age, sex, race, insurance status, fracture diagnosis, and visit characteristics were extracted from the EHR and used to asses differences in patient and visit characteristics associated with note labels. Analyses were performed with SAS (Cary, NC) version 15.2, R studio, and excel.

To evaluate the performance of each model on the binary outcome measure we evaluated the F1 score, sensitivity, specificity, positive and negative predictive value, and Area Under the Receiver Operating Characteristic Curve (AUC-ROC) metric. The AUC-ROC metric provides a single value that encapsulates the trade-off between the true positive rate and the false positive rate at various decision threshold levels. Model comparisons are displayed using ordered Box and Whisker plots where each box represents the interquartile range (IQR) for a specific model's AUC scores and data points represent outliers. To assess statistical significance between model performance, we conducted paired *t*-tests between models that shared identical data splits and unpaired *t*-tests (Welch's *t*-test) for comparisons involving the CNN model. Since the transformer-based and traditional machine learning models used identical data splits in each run, paired *t*-tests were appropriate for these comparisons. In contrast, due to the CNN model's different tokenization and processing pipeline, unpaired *t*-tests were necessary for comparing CNN with other models. A significance level of *p* < 0.05 was set.

## Results

### Progress note characteristics

The sample of 868 clinical notes came from patients treated by 123 physicians across 35 departments within one regional health system. The average age of the patient was 67.5 years of age and 80% of the sample were female patients. Patients were primarily white (91%) and enrolled in the Medicare system (64%). The notes came from fracture-related encounters ranging from the 2nd to the 22nd encounter after the index PHF visit, with a mean of 4, representing the 4th PHF-related encounter. The note lengths ranged from 981 to 15,297 characters with a mean length of 4,961 characters.

The sample was stratified into 465 notes labeled as treatment success and 403 notes labeled as treatment failure. Patients experiencing treatment success did not differ from those labeled as treatment failure in age, sex, or race. Additionally, treatment success notes did not differ in time since index visit, or the number of PHF-related encounters. However, notes labeled as treatment success had a higher proportion of Medicare (65.8% vs. 62.3%) and commercially insured patients (23.4% vs. 22.1%), compared to notes labeled as treatment failure. Additionally, treatment success notes had a lower proportion of surgically treated patients (16.3% vs. 24.3%) than notes labeled as treatment failure. Treatment success notes were also significantly shorter in length (4,673 vs. 5,291 characters). Table 2 contains sample characteristics by note label status.

**Table 2:** Sample characteristics by note label Status.

Patient and note characteristics	Total sample	Treatment success notes	Treatment failure notes	*P*-value
	*N* (%)	*N* (%)	*N* (%)	
	*N* = 868 (100)	*N* = 465 (53.4)	*N* = 403 (46.4)	
Patient Age, Mean (SD)	67.5 (14.9)	68.1 (15.1)	66.8 (14.7)	0.19
Patient Sex, N (%)				0.18
Male	175 (20.2)	86 (18.5)	89 (22.1)	
Female	693 (79.8)	379 (81.5)	314 (77.9)	
Patient Race, N (%)				0.68
White	791 (91.1)	427 (91.8)	364 (90.3)	
Black	46 (5.3)	23 (4.9)	23 (5.7)	
American Indian, Alaskan, or Hawaiian	4 (1.0)	3 (0.6)	1 (0.2)	
Hispanic	13 (1.5)	6 (1.3)	7 (1.7)	
Asian	4 (0.5)	2 (0.4)	2 (0.5)	
Other	10 (1.0)	4 (1.0)	6 (1.5)	
Insurance Provider				0.04
Medicare	557 (64)	306 (65.8)	251 (62.3)	
Medicaid	37 (4.2)	16 (3.4)	21 (5.2)	
Private	198 (23)	109 (23.4)	89 (22.1)	
Other	70 (8.1)	34 (7.3)	36 (8.9)	
Note Characteristics				
Days from Index, Mean (SD)	135 (79.9)	131.2 (72.0)	140.8 (87.8)	0.08
PHF-related encounter, Mean (SD)	4.4 (1.8)	4.4 (1.6)	4.5 (2.01)	0.26
Patient treated surgically, N (%)	174 (20)	76 (16.3)	98 (24.3)	<0.01
Note character length, Mean (SD)	4,961 (2,725)	4,673.4 (2,511.9)	5,294.1 (2,921.3)	<0.001

### Orthopaedic resident agreement in note labeling

Each orthopaedic resident was assigned and labeled 268 notes with overlap in note assignment across orthopaedic residents. Interrater agreement for classification of clinical notes was moderate [pairwise percent agreement = 75.31%, Fleiss' *κ* = 0.49 (95% CI: 0.30–0.68)].

### Model performance metrics

**Figure 2:**
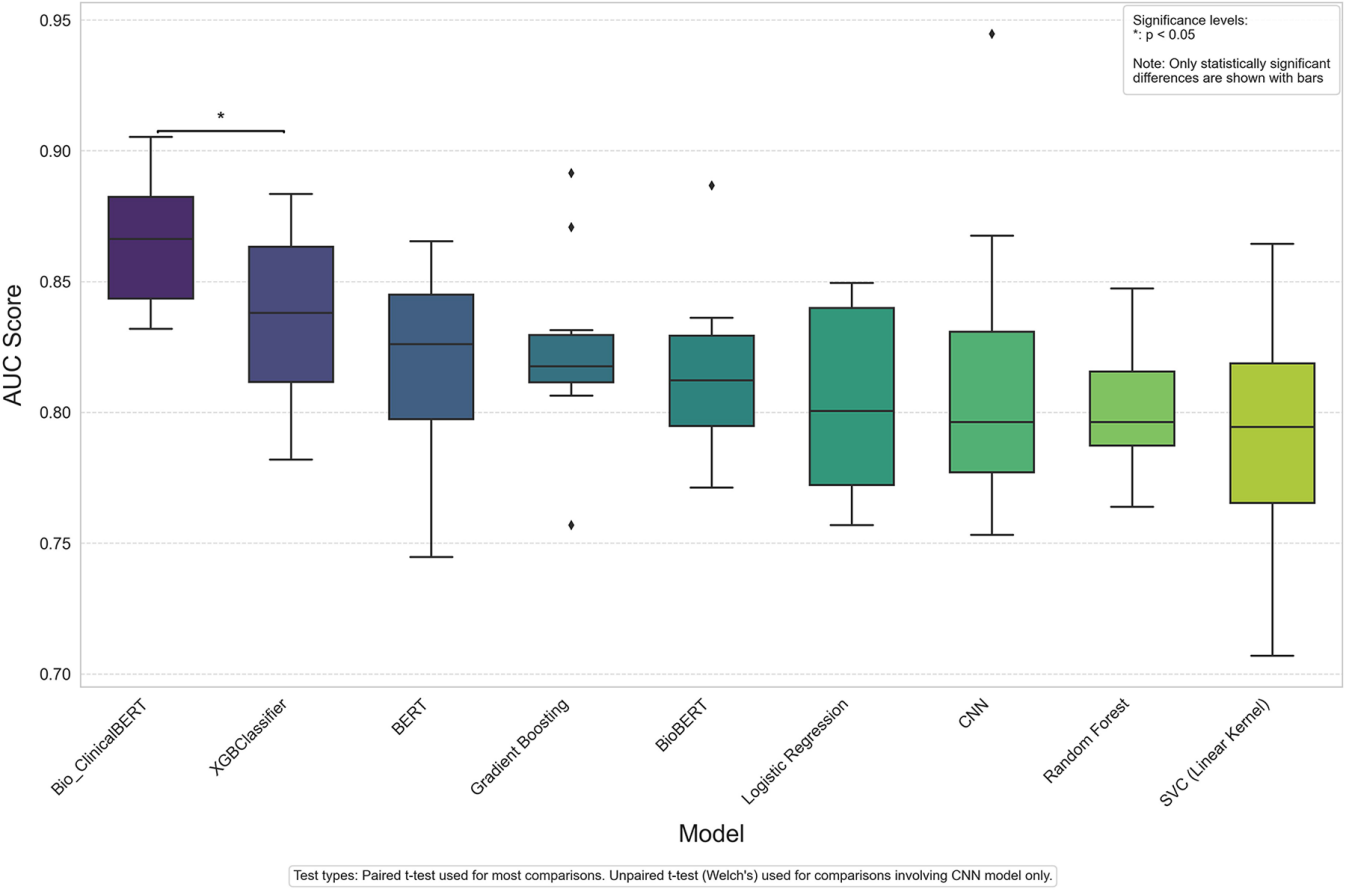
AUC scores across deep learning and machine learning models.

 Table 3 shows results for both machine and deep learning models explored in our analysis. The XGB Classifier model achieved the highest AUC score (0.84 ± 0.03) among the bag-of-words models and the Bio-ClinicalBERT model had the highest AUC (0.87 ± 0.04) among the deep learning models. Overall, the Bio-ClinicalBERT model had the highest performance (AUC = 0.87 ± 0.04) in correctly distinguishing between treatment success and failure notes. Table 3 shows complete performance metrics for machine and deep learning models and Figure 2 displays comparative model performance for all models.

**Table 3:** Performance metrics for Bag-of-word and deep learning models.

Model name	F1 score	Specificity	Sensitivity	Precision	NPV	AUC
Bag-of-words models						
Logistic regression	0.70 (±0.03)	0.64 (±0.03)	0.82 (±0.04)	0.72 (±0.02)	0.73 (±0.04)	0.80 (±0.03)
SVC	0.72 (±0.03)	0.60 (±0.03)	0.81 (±0.03)	0.71 (±0.02)	0.74 (±0.03)	0.79 (±0.03)
Random forest	0.72 (±0.03)	0.68 (±0.03)	0.80 (±0.02)	0.73 (±0.02)	0.73 (±0.03)	0.80 (±0.01)
Gradient boosting classifier	0.75 (±0.02)	0.70 (±0.02)	0.81 (±0.02)	0.75 (±0.02)	0.75 (±0.02)	0.82 (±0.01)
XGBClassifier	0.75 (±0.02)	0.70 (±0.06)	0.78 (±0.03)	0.75 (±0.03)	0.74 (±0.03)	0.84 (±0.03)
Deep learning models						
BERT	0.76 (±0.08)	0.76 (±0.04)	0.75 (±0.06)	0.79 (±0.05)	0.72 (±0.08)	0.83 (±0.05)
BioBERT	0.75 (±0.03)	0.71 (±0.07)	0.77 (±0.04)	0.77 (±0.05)	0.73 (±0.03)	0.81 (±0.03)
Bio-ClinicalBERT	0.77 (±0.04)	0.74 (±0.09)	0.80 (±0.04)	0.78 (±0.07)	0.76 (±0.02)	0.87 (±0.04)
CNN	0.73 (±0.09)	0.65 (±0.14)	0.77 (±0.07)	0.71 (±0.10)	0.69 (±0.11)	0.80 (±0.05)

## Discussion

This study is the first to develop a clinical text classifier capable of identifying whether a patient achieved treatment success following care for an acute PHF. Our results suggest that AI, more specifically, text classification models in combination with clinical note text can be used to differentiate patients with good and bad treatment outcomes. This finding is encouraging, and it suggests that routine clinical note content may offer a solution to the much-needed problem of outcome data availability in orthopaedic medicine. This AI approach could translate to producing a widely available treatment outcome measure, which could be used for comparative effectiveness research studies and for the development of clinical quality metrics (64) to improve the quality of orthopaedic care.

A crucial step in demonstrating that orthopaedic care achieves meaningful patient-specific improvement is to have outcome data available that closely measures if treatment was successful in achieving the outcome goals prioritized by patients. However, a leading challenge in outcome measurement remains that orthopaedic outcomes are highly individualized and patient specific. This fact undermines the value of even the industry-wide standard PROMs, as PROM overall scores often assume equal weighting of outcome dimensions for all patients. Yet, as an example, patients may more heavily value pain reduction over range of motion, which would not be accurately reflected in summary PROM scores. In contrast, our approach and resulting measure is highly flexible and responsive to each individual patient's treatment goals. Orthopaedic-related encounters are highly focused on each patient and how musculoskeletal conditions and treatment affect outcome dimensions and a patient's quality of life. Each encounter is naturally tailored to the patient and their unique musculoskeletal needs, and physicians record goal achievements or challenges that are revealed through the encounter discussion, exam and treatment planning.

The applications of AI and NLP are rapidly expanding in medicine (17, 32, 65, 66) and orthopaedic medicine (23, 34). Yet, our work is the first to use a text classifier to develop a binary measure of treatment success following care for an acute shoulder fracture. Recent studies have used similar methods to achieve related tasks. For example, Humbert-Droz et al., developed a NLP pipeline to extract documented PROMs from the EHR for patients with rheumatoid arthritis (24), and Zanotto et al., used NLP models to identify patient characteristics and health status PROMs (21). However, these studies differ from our approach in that they are still dependent upon the collection of PROM data during clinical care. Our approach offers advantages in that it does not require any additional data collection efforts on behalf of physicians, staff, or practice managers, as we know there are a myriad of challenges in collecting PROMs that may never be eliminated (10). Our approach is flexible and responsive with respect to the natural language typically recorded during the clinical encounter.

Our sample contained clinical notes from office visits for PHF-related care ranging from over 123 physicians across 35 departments within a regional health system. This finding signals that patients are receiving follow-up care for their PHFs from many different providers. While many of these visits were with an orthopaedic provider, this is an elderly patient population that regularly seeks care from primary care. Therefore, the diversity in providers and departments in our sample spans clinical notes authored by both orthopaedic and primary care physicians. This might signal that our approach is robust to notes beyond those authored just by orthopaedic physicians, to also include a variety of physicians that may discuss healing following orthopaedic treatment. Although there were not many differences observed between the characteristics of success and failure notes, we did find that treatment success notes were significantly shorter in length (4,673 vs. 5,291 characters) compared to treatment failure notes. This is not surprising, as good outcomes can be discussed and recorded more quickly and typically don't require ongoing treatment and related discussion and documentation. However, clinical documentation can be highly varied and more robust patient and visit characteristics should be assessed in future work to explore their relationship with clinical documentation and patient outcomes.

We found that of the models we tested, the Bio-ClinicalBERT model had the highest performance (AUC = 0.87 ± 0.04) in correctly distinguishing between treatment success and failure notes. This finding was consistent with prior research, which has illustrated Bio-ClinicalBERT's enhanced capability in comprehending intricate medical terminologies and thematic elements inherent to clinical documentation compared to other models. In a similar study, Lee et al. also found that Bio-ClinicalBERT models performed well when identifying documented goals-of-care discussions within the EHR (22).

This work was the first study to develop a clinical text classifier capable of identifying whether a patient achieved treatment success following care for an acute PHF. Our ongoing work will continue to refine our approach to how to use clinical progress note content to form assessments of patient outcomes following orthopaedic treatment. Beyond validation and moving toward use in clinical practice, we envision the next step would be to consider integrating this measure with the EHR to support clinical decision support (CDS) use and quality reporting at the health system level. Development of this measure provides science at scale which enables unlimited comparative effectiveness studies across orthopaedic treatments and future tailored treatment decision making through CDS use to improve individual and population health.

This work is not without limitations. This was a relatively small feasibility study to assess if text classifiers could be applied to clinical notes to generate a measure of orthopaedic treatment success. Although we were successful in achieving an outcome measure with a high level of accuracy, we used a relatively small sample of patients with one shoulder condition. The patient sample we used lacked diversity, being primary female and white, and model performance should be assessed in more diverse patient samples to ensure unbiased model performance among patient subsets. Additionally, clinical note structure can vary significantly across physicians, medical specialties, and health systems. To inform the generalizability of our work, we must test our approach using multi-institutional data across a range of orthopaedic providers with variable documentation practices. Although we expect our approach would be generalizable to other orthopaedic conditions, we have not explored our methods in orthopaedic conditions beyond PHF. Future work will focus on applying our approach to acute and chronic orthopaedic conditions across adult and pediatric populations. Furthermore, the transformer-based models we adopted including BERT, BioBERT, and ClinicalBioBERT, are relatively limited in their parameter scope and the breadth of training data compared to more recent advancements in large language models (LLMs). These newer models, which boast significantly more parameters and have been trained on substantially larger datasets, may offer improved accuracy and better generalizability across diverse medical texts and clinical conditions. Exploring the application of LLMs in future studies could address some of the current limitations and provide a more robust tool for clinical text analysis.

As a next step for this work, we plan to assess the validity of our newly developed measure using PROMs or prospective patient follow-up. There are mixed reports of the accuracy of clinical note content, therefore we must evaluate if our measure accurately reflects a state of patient improvement or decline our new measure is reporting. However, multiple studies have found that healthcare professionals produce accurate documentation for concrete and overt symptoms, such as range of motion and impaired physical functioning (67–70). Therefore, because of the highly focused nature of orthopaedic encounters and symptoms, we believe that clinical note content remains a valuable and valid source of data from which to develop an indicator of treatment success for patients (17, 19, 38). Lastly, although we engaged patients in the development of outcome labels and the definition of treatment success, we ultimately used the physician perspective to develop the gold standard dataset labels of treatment success or failure. It is recognized that patients and physicians may differ in their definition of treatment success, therefore future work will explore incorporating patient perspectives into the definition of treatment success.

## Conclusions

We believe the development of an AI-based measure of treatment success using clinical notes may represent a paradigm shift in how outcomes are collected in orthopaedic medicine. Orthopaedic-related healthcare encounters are highly focused on the ways in which musculoskeletal treatments affect outcome priorities such as pain, range of motion, and patient's quality of life. This method, if widely adopted and implemented on clinical note content stored in the EHR could translate to widely available success measures which could be used to for comparative effectiveness research studies and for the development of clinical quality metrics (64) to improve the quality of orthopaedic care.

## Data Availability

The data used in this analysis is not readily available because patient identifiers are contained in the dataset and they must remain stored in a HIPAA compliant location. Requests to access the datasets should be directed to sbf@clemson.edu.
